# Platinum versus immunotherapy for early resectable non-small cell lung cancer: A protocol for systematic review and meta analysis

**DOI:** 10.1097/MD.0000000000022349

**Published:** 2020-10-23

**Authors:** Zhangwei Tong, Fei Luo, Xiaojie Yang, Mingqiang Kang, Jiangbo Lin

**Affiliations:** Department of Thoracic Surgery, Fujian Medical University Union Hospital, Fuzhou, China.

**Keywords:** chemotherapy, immunotherapy, non-small cell lung cancer, platinum chemotherapy, radiotherapy

## Abstract

**Background:**

Lung cancer is one of the most common malignant tumors. Non-small cell Lung cancer (NSCLC) accounts for about 85% of the total lung cancer. For patients with resectable early NSCLC, conventional postoperative adjuvant therapy can significantly prolong the overall survival of patients and reduce the risk of tumor recurrence. With the emergence and maturity of molecular targeted therapy and immunotherapy, the strategy of postoperative chemotherapy for lung cancer patients has changed greatly. To evaluate the efficacy of postoperative chemotherapy (platinum based chemotherapy and immunotherapy) with or without radiotherapy for NSCLC patients, we will conduct a systematic review and meta-analysis of published or unpublished randomized controlled trials.

**Methods:**

We will search Pubmed (Medline), Embase, Google Scholar, Cancerlit, and the Cochrane Central Register of Controlled Trials for related studies published without language restrictions before June 20, 2021. Two review authors will search and assess relevant studies independently. Randomized controlled trials and quasi-randomized controlled trials studies will be included. we will perform subgroup analysis in different methods of postoperative adjuvant therapy for patients with resectable early NSCLC. Because this study will be based on published or unpublished records and studies, there is no need for ethics approval. INPLASY registration number: INPLASY202080064.

**Results:**

The results of this study will be published in a peer-reviewed journal.

**Conclusion:**

This study will compare the efficacy of platinum chemotherapy and immunotherapy in patients with resectable early NSCLC. Since the large sample randomized trials that meet the inclusion criteria of this study may be inadequate, we will consider incorporating some high quality small sample related tests, which may lead to heterogeneity and affect the reliability of the results.

## 1 Introduction

Globally, lung cancer is the second most common malignancy. It is highly malignant and is the leading cause of cancer-related death.^[1]^ Non-small cell lung cancer (NSCLC) accounts for about 85% of the newly diagnosed lung cancer cases each year, which is the most common pathological type of lung cancer.^[2,3]^

Surgical resection of early resectable NSCLC offers potential therapeutic opportunities for patients considered to be the best treatment.^[4]^ The 5-year survival rate of patients with pathological stage Ia to IIIa NSCLC after surgery is 73% to 25%.^[5,6]^ However, there is no suitable treatment for the recurrence or metastasis of NSCLC. The 5-year survival rate of the patients can be increased by about 5% after the operation.^[7–9]^

However, adjuvant radiotherapy is not recommended for postoperative adjuvant therapy of early-stage NSCLC because it can significantly increase the mortality rate of patients by about 7% in 2 years.^[6,10,11]^ With the emergence of molecular targeted therapy and immunotherapy, postoperative adjuvant therapy has become diversified.^[12]^ At present, although there is not enough evidence to support that PD1/PD-L1 inhibitors can effectively improve postoperative outcomes, more high-quality clinical trials are underway or have been published.

Objective to evaluate the efficacy of postoperative chemotherapy (platinum based chemotherapy and immunotherapy) in patients with or without radiotherapy for NSCLC, and obtain reliable evidence. We will conduct a systematic review and comprehensive analysis of published or unpublished relevant trials, and then compare these with the postoperative treatment methods of patients with NSCLC, so as to provide reference for clinicians and formulate the best treatment strategy.

## 2 Objective

We will evaluate the efficacy of postoperative adjuvant therapy (platinum based chemotherapy and immunotherapy) with or without radiotherapy for patients with NSCLC.

## 3 Methods

This protocol is conducted according to the preferred reporting items for systematic review and meta-analysis protocols statement.^[13]^ We will report the results of this systematic review and meta-analysis adhere to the preferred reporting items for systematic reviews and meta-analysis guidelines.^[14]^ This protocol has been registered in the INPLASY network (registration number: INPLASY202080064).

### 3.1 Patient and public involvement

This study will be based on published or unpublished studies and records and will not involve patients or the public directly.

### 3.2 Eligibility criteria

#### 3.2.1 Types of studies

Randomized controlled trials and quasi-randomized controlled trials published or unpublished will be included, which have been completed and compared postoperative platinum-base chemotherapy versus immunotherapy for patients with NSCLC.

#### 3.2.2 Types of participants

The participants will be adults diagnosed with resectable early NSCLC histologically or cytologically confirmed who were treated with platinum-based chemotherapy, or immunotherapy after surgery. No restrictions on ethnicity, sex, education, and economic status will be applied.

#### 3.2.3 Types of interventions

According to the means of postoperative chemotherapy for patients with resectable early NSCLC, the trials included will be divided into the following categories.

- immunotherapy versus molecular targeted therapy
- immunotherapy versus anti-angiogenic agents
- postoperative platinum-base chemotherapy versus molecular targeted therapy
- platinum-based chemotherapy versus anti-angiogenic agents
- platinum-based chemotherapy versus immunotherapy

#### 3.2.4 Types of outcome measures

##### 3.2.4.1 Primary outcomes

The primary outcomes will be postoperative overall survival of patients with resectable early NSCLC who were treated with postoperative chemotherapy.

##### 3.2.4.2 Secondary outcomes

We will assess the 5-year survival, median survival, recurrence-free survival, quality of life, and adverse events or complications of patients with resectable early NSCLC who were treated with postoperative chemotherapy.

### 3.3 Information sources

We will search Pubmed (Medline), Embase, Google Scholar, Cancerlit, and the Cochrane Central Register of Controlled Trials for related studies published before June 20, 2021 without language restrictions.

### 3.4 Search strategy

We will use the relevant keywords or subject terms adhered to Medical Subject Heading terms to search for eligible studies in the electronic databases which were mentioned above without language restrictions. The Pubmed search strategies are shown in Table 1.

**Table 1:**
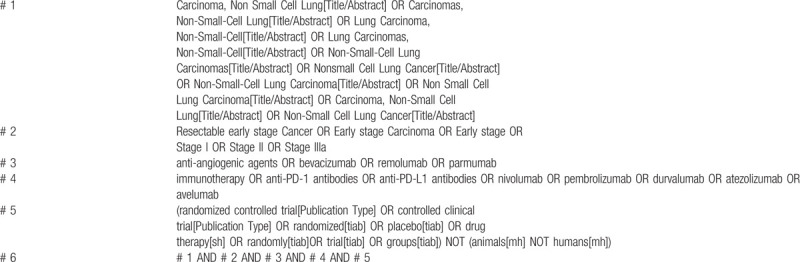
Pubmed search strategies.

### 3.5 Data collection and analysis

We will utilize the measures described in the Cochrane Handbook for Systematic Reviews of Interventions to pool the evidence.^[15]^

#### 3.5.1 Study selection

Two reviewers (ZWT, FL) will investigate each title and abstract of all literatures searched independently and identify whether the trials meet the inclusion criteria as designed and described in this protocol. Two authors (ZWT, FL) will in duplicate and independently screen the full text of all potential eligible studies to exclude irrelevant studies or determine eligibility. The 2 reviewers will list all the studies included and document the primary reasons of exclusion for studies that do not conform to the inclusion criteria. Disagreements between the 2 authors will be resolved by discussing with the third author (XJY), if necessary, consulting with the fourth author (JBL). We will show the selection process in details in the preferred reporting items for systematic reviews and meta-analysis flow chart.

#### 3.5.2 Data extraction and management

The 2 authors (ZWT, FL) will extract the following data independently from the studies included.

- Study characteristics and methodology: publication date, the first author, country, randomization, study design, periods of data collection, follow-up duration, total duration of study, withdrawals, and so on.
- Participant characteristics: gender, age, tumor stage, pathology diagnosis, ethnicity, performance status, history of smoking, pathologic tumor size, inclusion criteria, and so on.
- Interventions: type of operation, extent of resection, therapeutic means, drugs, dosage, modality, frequency of administration, and so on.
- Outcome and other data: overall survival, 5-year survival, median survival, disease-free survival, 95% confidence intervals (CIs), recurrence time, quality of life, adverse events, complications, and so on.

We will record all the date extracted in a pre-designed table and consult the first author of the trial by e-mail before determining eligibility, if the reported data of which are unclear or missing.

### 3.6 Assessment of risk of bias in included studies

Two authors (ZWT, FL) will use the Cochrane Handbook for Systematic Reviews of Interventions to assess the risk of bias of each study included independently based on the following ranges: random sequence generation (selection bias); allocation concealment (selection bias); blinding of participants and personnel (performance bias); blinding of outcome assessment (detection bias); incomplete outcome data (attrition bias); selective outcome reporting(reporting bias); other bias.^[13]^ Each domain will be assessed as high, low or uncertain risk of bias. The results and details of assessment will be reported on the risk of bias graph.

### 3.7 Data analysis

The data will be synthesized by Review Manager 5.3 software. We will conduct a systematic review and meta-analysis only if the data gathered from included trials are judged to be similar enough to ensure a result that is meaningful. The Chi^2^ test and *I*^2^ statistic will be used to assess statistical heterogeneity among the trials included in matched pairs comparison for standard meta-analysis. The random effect model will be applied to analyze the data, if there is substantial heterogeneity (*P* < .1 or *I*^2^ statistic > 50%) and the trials will be regarded to be obvious heterogeneous. Otherwise, we will utilize fixed effect model to analyze the data. Mantel–Haenszel method will be adopted to pool of the binary data. The results will be reported in the form of relative risk between 95% CI of the date. The continuous data will be pooled by inverse variance analysis method and the results will be shown in the form of standardized mean difference with 95% CI of the date.

#### 3.7.1 Subgroup analysis

If there is high heterogeneity (*I*^2^ statistic > 50%) and the data are sufficient, subgroup analysis will be conducted to search potential causes of heterogeneity. Subgroup analysis will be performed in different methods of postoperative adjuvant therapy, ethnicity, history of smoking, tumor stage, and type of operation.

#### 3.7.2 Sensitivity analysis

Sensitivity analysis will be conducted to assess the reliability and robustness of the aggregation results via eliminating trials with high bias risk.

### 3.8 Publication bias

If there are 10 or more than 10 trials included, we will construct a funnel plot and use Egger test to assess publication bias. If reporting bias is suspected, we will consult the study author to get more information. If publication bias does exist, we will apply the fill and trim method to analyze publication bias in the trials.^[16]^

### 3.9 Evidence evaluation

We will evaluate all the evidence according to the criteria of grading of recommendations, assessment, development, and evaluation (imprecision, study limitations, publication bias, consistency of effect, and indirectness bias). The quality of all evidence will be evaluated as 4 levels (high, moderate, low, and very low).^[17]^

## 4 Discussion

The 5-year survival rate of NSCLC with pathological stage IA to IIIA was 73% to 25%. There was no suitable treatment for recurrence and metastasis of NSCLC. Postoperative adjuvant therapy is a necessary means to improve the overall survival rate and quality of life of patients with early resectable NSCLC. With the emergence of molecular targeted therapy and immunotherapy, postoperative adjuvant therapy has become diversified. It is a challenge for clinical oncologists to integrate different therapies to treat early NSCLC. Therefore, it is necessary to explore the diversity of adjuvant therapy to determine the optimal treatment strategy for each resectable NSCLC patient. The purpose of this study is to provide a reliable basis for integrating different treatment methods and formulating the optimal treatment strategy for early NSCLC.

## Author contributions

**Conceptualization:** Zhangwei Tong, Fei Luo, Mingqiang Kang.

**Data curation:** Zhangwei Tong, Fei Luo, Mingqiang Kang.

**Formal analysis:** Zhangwei Tong, Fei Luo, Xiaojie Yang, Mingqiang Kang.

**Funding acquisition:** Zhangwei Tong.

**Investigation:** Zhangwei Tong, Jiangbo Lin.

**Methodology:** Zhangwei Tong.

**Project administration:** Zhangwei Tong.

**Resources:** Mingqiang Kang.

**Software:** Mingqiang Kang.

**Supervision:** Jiangbo Lin.

**Validation:** Jiangbo Lin.

**Visualization:** Jiangbo Lin.

**Writing – review & editing:** Jiangbo Lin.
